# Methods and processes to develop and deliver a theory-informed education program for sustained behaviour change in emergency nursing

**DOI:** 10.1371/journal.pone.0323115

**Published:** 2025-06-06

**Authors:** Julie Considine, Belinda Kennedy, Margaret Murphy, Margaret Fry, Ramon Z. Shaban, Kate Curtis

**Affiliations:** 1 School of Nursing and Midwifery and Centre for Quality and Patient Safety Research in the Institute for Health Transformation, Deakin University, Geelong, Victoria, Australia; 2 Centre for Quality and Patient Safety Research, Eastern Health, Box Hill, Victoria, Australia; 3 Faculty of Medicine and Health, The University of Sydney Susan Wakil School of Nursing and Midwifery, Camperdown, New South Wales, Australia; 4 Western Sydney Local Health District, North Parramatta, New South Wales, Australia; 5 University of Technology Sydney Faculty of Health, New South Wales, Australia; 6 Northern Sydney Local Health District, New South Wales, Australia; 7 Sydney Institute for Infectious Diseases, Faculty of Medicine and Health, The University of Sydney, Camperdown, New South Wales, Australia; 8 New South Wales High Consequence Infectious Diseases Specialist Service, NSW Biocontainment Centre, Western Sydney Local Health District, Westmead, New South Wales, Australia; 9 Emergency Services, Illawarra Shoalhaven Local Health District, Wollongong Hospital, Wollongong, New South Wales, Australia; Mersin University: Mersin Universitesi, TÜRKIYE

## Abstract

**Background:**

HIRAID® (**H**istory including **I**nfection risk, **R**ed flags, **A**ssessment, **I**nterventions, **D**iagnostics, communication and reassessment) is an evidence-based framework that supports emergency nurses to optimise safety, quality, and patient experience of care. HIRAID® was the intervention in a modified stepped-wedge cluster randomised controlled trial (SW-cRCT) in a convenience sample of 29 Australian emergency departments (Australian New Zealand Clinical Trials Registry: ACTRN12621001456842). The aim of this paper is to describe the methods and processes used to develop and deliver a theory-informed education program to support behaviour change during HIRAID® implementation.

**Methods:**

The HIRAID® education program was developed using: i) existing HIRAID® research using the Behaviour Change Wheel and Theoretical Domains Framework to identify enablers and barriers to HIRAID® use; ii) application of educational pedagogical theoretical frameworks (constructive alignment, backwards design, scaffolded learning); Bloom’s taxonomy of educational objectives, and active and collaborative learning; iii) Australian standards related to safety, quality, clinical governance, and emergency nursing; and iv) behavioural diagnostic data from study sites (n = 670 nurses).

**Results:**

HIRAID® education program consisted of HIRAID® Provider and Instructor Courses and was delivered using a ‘train-the-trainer’ model. Fifteen HIRAID® Instructor Courses were held from February 2021 to March 2023 with 162 participants, and at November 2023 over 1300 emergency nurses had completed the HIRAID® Provider Course.

**Conclusions:**

The theory-informed approach to the HIRAID® education program enabled development of a structured program and delivery in the dynamic and complex emergency department environment. The approach reported in this paper provides a blueprint for other researchers aiming to change behaviours in complex settings.

## Introduction

Evidence-informed practice improves patient outcomes and optimises healthcare resource use [1], however, it takes an average of 17 years to implement research evidence into practice [2]. Sustained implementation of evidence-informed practice is particularly challenging for complex and dynamic clinical environments, such as emergency departments (EDs) [3,4]. Emergency nurses care for undiagnosed, undifferentiated patients of all ages, with varying degrees of illness or injury severity, and who span all healthcare specialities [5]. The ED is also an environment with unpredictable workloads, overcrowding, significant time pressures, constant interruptions, and high levels of uncertainty [5]. A scoping review of 25 studies of education interventions intended to change emergency nurses’ clinical practice behaviours showed that few studies were underpinned by behaviour change theory or educational pedagogy, and few clearly identified the cognitive domains of interest [6] which limits their success.

**Fig 1:**
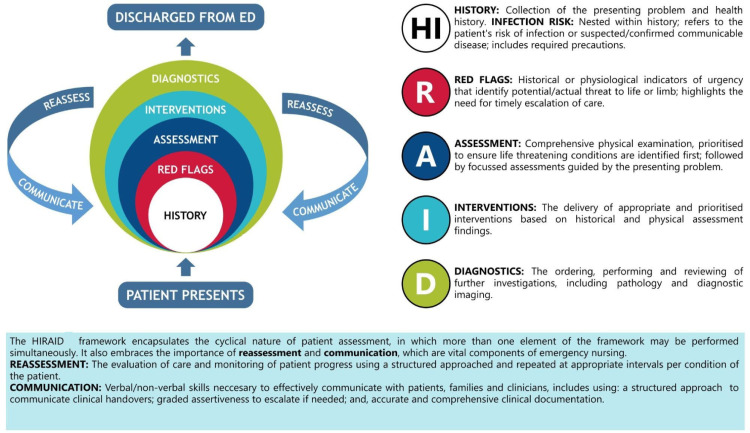
HIRAID® emergency nursing assessment framework.

Patient safety in the ED is contingent on emergency nurses’ accurate assessment, interpretation of clinical data, intervention and escalation of care of deteriorating patients [5,7]. HIRAID® (**H**istory including **I**nfection risk, **R**ed flags, **A**ssessment, **I**nterventions, **D**iagnostics, communication and reassessment) is an evidence-based emergency nursing framework that supports emergency nurses to optimise safety, quality, and patient experience of emergency care (Fig 1). In a high-fidelity simulation study, HIRAID® improved quality of emergency nursing assessment, recognition and escalation of indicators of urgency, and frequency of ‘patient’ reassessments [8]. During real-world testing, HIRAID® was considered acceptable, and appropriate for use in emergency care contexts [9] and improved quality of documentation of emergency nursing assessments [10]. Further HIRAID® implementation reduced clinical deterioration related to emergency nursing care for ED patients requiring hospital admission from 21% to 8% [11] resulting in significant cost savings (AUD$1.9million) [12,13].

At the time of writing this paper, HIRAID® was the intervention in a modified stepped-wedge cluster randomised controlled trial (SW-cRCT) in a convenience sample of 29 EDs in New South Wales and Victoria, Australia (2021–2025) [14]. Critical to this SW-cRCT is HIRAID® dose, which is reliant on the effectiveness of implementation and intervention fidelity [14]. Although evidence-informed theory and targeted behaviour change techniques increase likelihood of successful and sustained change, achieving effective and sustained behaviour change in EDs is challenging given the complex and dynamic nature of the environment. For example, a large scale c-RCT in 26 Australian EDs aiming to improve ED care of patients with stroke did not result in the same improvements in care seen in 19 Australian stroke units, despite having an evidence-based, theory-informed implementation strategy [15,16]. A post-hoc qualitative study (n = 28) yielded three major themes: readiness to change; fidelity to the protocols; and boundaries of care [17]. The data showed that high levels of clinician engagement, the volume of emergency nurses requiring education and clinical support, beliefs about the fidelity of evidence, difficulties operationalising seemingly straight-forward protocols and issues related to role and professional identity impeded protocol uptake [17].

**Fig 2:**
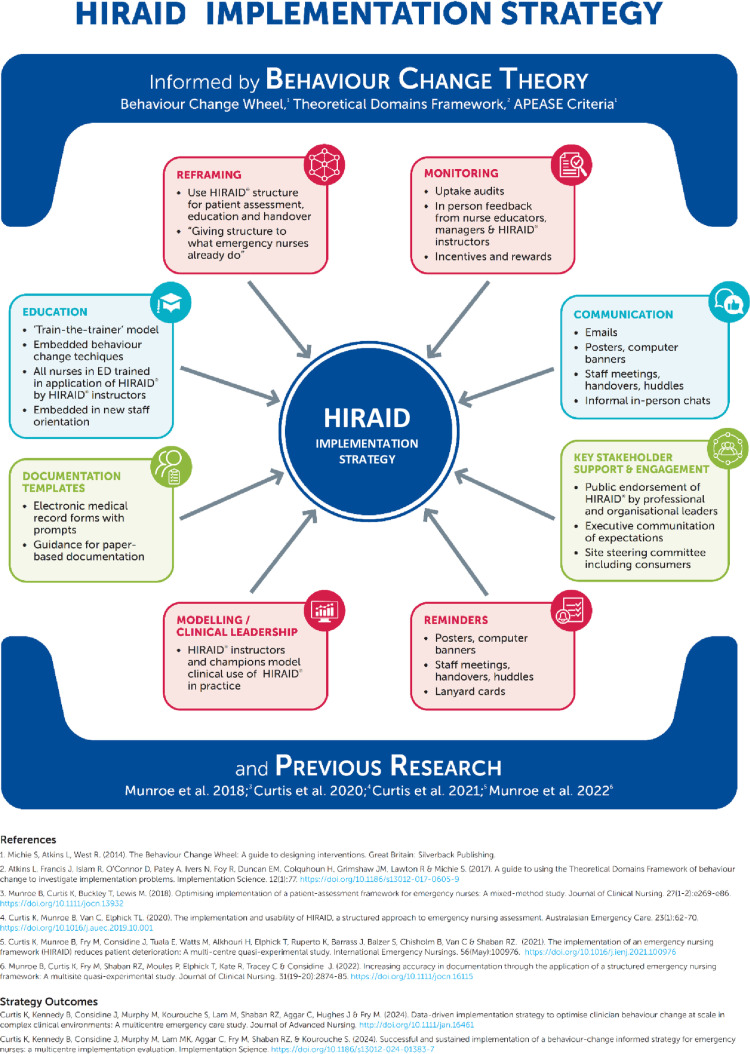
HIRAID® implementation strategy.

Education was a major component of HIRAID® testing in simulated [8,18,19] and real-world contexts [9]. Thus, the educational approach was a core component of the HIRAID® SW-cRCT implementation strategy [20] (Fig 2). Behavioural diagnostics from a feasibility study conducted in three Australian EDs in 2017 showed that the educational resources perceived by nurses as most helpful in enabling use of HIRAID® were written resources (49%), online learning modules (55%), and formal training using workshops (70%) [9]. However, education alone is known to have a limited impact on behaviour change [1]. A 2023 scoping review of 25 studies of the effect of education interventions on emergency nurses’ clinical practice behaviours showed that few addressed elements of behaviour change theory or targeted cognitive domains [6].

### Aim

The aim of this paper is to describe the methods and processes used to develop and deliver a theory-informed education program to support behaviour change in emergency nursing. The desired behaviour change was use of the HIRAID® emergency nursing framework in the context of a modified SW-cRCT.

## Methods

The purpose of this methods section is to describe *the development* of the HIRAID® education program that occurred in four phases. In Phase 1, a critical appraisal of published HIRAID® research using the Behaviour Change Wheel and Theoretical Domains Framework to identify known enablers and barriers to HIRAID® use was conducted. Educational pedagogical theoretical frameworks (constructive alignment, backwards design, scaffolded learning; Bloom’s taxonomy of educational objectives; and active and collaborative learning) were applied to the development of the HIRAID® education program in Phase 2. In Phase 3, alignment with Australian standards related to safety, quality, clinical governance, and emergency nursing occurred. Finally, in Phase 4 behavioural diagnostic data from study sites (n = 670 nurses) was used to identify context specific enablers and barriers to HIRAID® use and inform consultation with end users (emergency nurses, nurse educators, nurse managers, and nursing executives). The study setting is also described to provide context for Phase 4. Ethics approval for Phase 4 was obtained from Greater Western Human Research Ethics Committee (2020/ETH02164) for the New South Wales sites, and from Royal Brisbane & Woman’s Hospital Human Research Ethics Committee (2021/QRBW/80026) for the Victorian sites.

### Phase 1: Critical appraisal of published HIRAID® research

The first phase of development of the HIRAID® education program was to critically examine the published research related to HIRAID® implementation, with a particular focus on known enablers and barriers to HIRAID® use, and previously successful implementation strategies. Four studies were identified [8,9,18,19] and all used the Behaviour Change Wheel [21] including the Theoretical Domains Framework (TDF) [26,27] as a theoretical framework.

**Fig 3:**
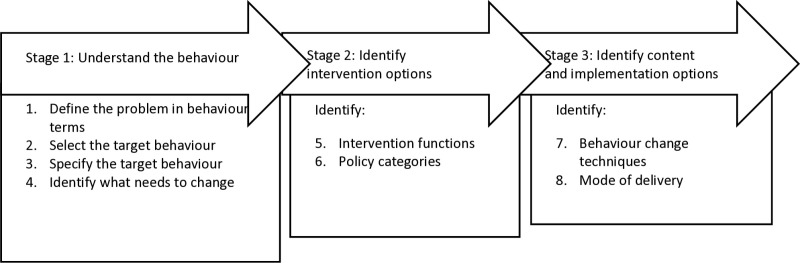
Behaviour change intervention design process.

Behaviour Change Wheel is a synthesis of 19 behaviour change frameworks, has three stages and eight steps of behaviour change intervention design [21] (Fig 3), and is underpinned by the premise that behaviour is dependent on capability, opportunity, motivation (referred to as COM-B) [21,22]. The Behaviour Change Wheel details nine intervention functions that leverage personal agency and external influences to optimise COM-B, seven policy categories that enable the intervention functions, and 93 behaviour change techniques that inform the components of an intervention designed to change behaviour [21]. The TDF is a synthesis of behaviour change theories [23,24] embedded in the Behaviour Change Wheel [21]. The TDF consists of 14 domains (knowledge; skills; social/professional role and identity; beliefs about capabilities; optimism; beliefs about consequences; reinforcement; intentions; goals; memory, attention and decision processes; environmental context and resources; social influences; emotion and behavioural regulation) linked to the COM-B components and intervention functions [21]. Given the intent of the HIRAID® education program was to support HIRAID® use in clinical practice (and thus change emergency nurses’ behaviour), the Behaviour Change Wheel [21] and Theoretical Domains Framework (TDF) [23,24] were appropriate to understand drivers of behaviour, barriers and enablers of behaviour, and embed behaviour change techniques into the HIRAID® education program

The first study was a simulation study (2013) involving 38 registered nurses from five New South Wales EDs [8,18]. Participants attended a 4-hour interactive education workshop, between pre and post participation in immersive simulation scenarios based on real ED presentations and using professional actors [8,18]. The workshop content was: introduction to HIRAID® components, documentation of assessment findings using HIRAID®, effective communication using ISBAR (Introduction, Situation, Background, Assessment, Recommendations/Requests), escalation of care to medical officers using graded assertiveness, and application of HIRAID® in the clinical setting [8,18]. The workshop was designed using principles of experiential learning (learning is generated from experience and reflection) and transformative learning (acknowledges the importance of changing beliefs and attitudes to successfully transform behaviour) [8,18]. The workshop was delivered using videos of nurses performing patient assessments with and without HIRAID®, PowerPoint presentations, case studies with interactive group discussions and role play.

Participants from the 2013 simulation study also completed written surveys and a focus group related to enablers and barriers to future implementation of HIRAID® into emergency nursing practice [19]. The Capability-Opportunity-Motivation-Behaviour (COM-B) components of the Behaviour Change Wheel, and Theoretical Domains Framework were used to analyse the survey and focus group data [19]. The enablers related to education and training were nurses’ clinical experience, existing patient assessment knowledge, introduction to HIRAID® within three months of commencing in ED, and learning opportunities within the ED [19]. The barriers related to education and training in relation to the application of HIRAID® were variability in nurses’ patient assessment skills, lack of knowledge of HIRAID®, uncertainty about application of HIRAID® in clinical practice, decline in knowledge of HIRAID® components over time, and the need for practice to develop confidence to use HIRAID® in their clinical practice [19]. The Behaviour Change Techniques that supported the education component of a future HIRAID® implementation strategy were related to instruction, demonstration and feedback. Modes of delivery were case-based eLearning, practical training using simulation exercises, and communication workshops [19].

In November 2017, HIRAID® was implemented in four EDs in one New South Wales Local Health District [9–11]. As part of the implementation strategy, the education component included an online learning module, workshops using a ‘train the trainer’ model, and simulation exercises [9]. The education approach was based on the barriers and enablers assessment by Munroe et al. [19] during the 2013 simulation study and delivery was informed by the APEASE criteria (Affordability, Practicability, Effectiveness and cost-effectiveness, Acceptability, Side-effects/safety and Equity) [21]. Real-world testing showed that HIRAID® was acceptable, feasible, practical and appropriate for use in the ED [9]; reduced treatment delays, delay or failure to escalate care of patients with abnormal vital signs, and isolated nursing-related causal factors of deterioration [11]; reduced clinical deterioration (rapid response system calls) in first 72 hours of hospital admission for patients admitted via the ED [11], and improved the accuracy of nurses’ documentation [10].

### Phase 2: Application of educational pedagogical theoretical frameworks

The second phase of HIRAID® education program development was to draw on additional educational pedagogical theoretical frameworks known to enhance learning outcomes (constructive alignment, [25–27] backwards design [28], scaffolded learning [29], and Bloom’s taxonomy of educational objectives [30] and active and collaborative learning [31]).

Constructive alignment [25–27] and backwards design [28] are outcomes-driven approaches focused on what participants should be able to do and what is the expected standard. Scaffolding refers to the connection of new knowledge to existing knowledge, and is achieved by purposeful design of learning outcomes, resources, and activities so that learners achieve successful outcomes in learning core concepts before moving on to more complex concepts [32]. Scaffolding is critical for knowledge retrieval [33]. In emergency nursing practice, knowledge retrieval occurs under significant time pressure and conditions of uncertainty, so deep learning is essential to embedding knowledge stored in short-term memory to long-term memory, enabling effective knowledge retrieval when required.

Clear and measurable intended learning outcomes, that increase in complexity and build on each other (scaffolding [29]), are critical to sound education design. Bloom’s taxonomy of educational objectives [32] spans six major cognitive domains (knowledge, comprehension, application, analysis, synthesis, and evaluation) that move learners from lower order thinking to higher order thinking [28]. Despite many educational interventions in emergency nursing aiming to increase knowledge or skills to effect change in emergency nurses’ clinical practice behaviours, less than half address the cognitive domains of analysis, synthesis, and very few address evaluation [6].

Intended learning outcomes were developed using Bloom’s taxonomy of educational objectives for each component of HIRAID® so facilitators and participants had clarity about what they are trying to achieve [30]. Further, deliberate design to cover all six cognitive domains meant that the HIRAID® education program could be tailored to participants’ scope of practice and level of clinical progression. The deliberate movement of participants from simple lower order thinking with low level uncertainty to complex higher order thinking with higher levels of uncertainty [32] aligns with emergency nurses’ typical trajectory of clinical progression, in which the acuity and complexity of the patients for whom they care increases [34,35]. Requisite to this clinical progression, is increasing complexity of knowledge, skills, and decision-making [34,35], thus the HIRAID® learning resources were deliberately sequenced to ensure scaffolded learning [29].

Active and collaborative learning is a student-centred approach to education delivery that empowers students to actively shape their learning experiences [31]. The ‘teacher’ takes on a facilitative role to foster collaboration, exploration of concepts, finding solutions, and providing meaningful feedback [36]. Active and collaborative learning increases student engagement, which improves learning outcomes, but also develops communication and teamwork skills, problem solving and critical thinking skills, and professional behaviours [37], all of which are essential to patient safety in the ED [7]. This pedagogical approach is well aligned to emergency nursing as it facilitates deep learning, which is vital to integration of new knowledge with experiential learning to manage complex, dynamic and safety-critical clinical problems [38].

### Phase 3: Alignment with Australian standards related to safety, quality, clinical governance, and emergency nursing

In Australia, all publicly funded hospitals are accredited against eight National Safety and Quality Health Service Standards [39]: i) Clinical Governance; ii) Partnering with Consumers; iii) Preventing and Controlling Infections, iv) Medication Safety; v) Comprehensive Care (includes falls, pressure injuries, hydration, nutrition, delirium and cognitive impairment, self-harm and suicide, violence and aggression, restrain and seclusion); vi) Communicating for Safety; vii) Blood Management; and viii) Recognising and Responding to Acute Deterioration [39]. These Standards aim to protect the public from harm, improve the quality of health service provision, and provide a nationally consistent statement about expectations of care [39]. Therefore, compliance with Australia’s National Safety and Quality Health Service Standards [39] is critical to safety and quality of health care, consistency of care delivery, and hospital accreditation. HIRAID® was always grounded in safety and quality of care but overtly aligning HIRAID® to the National Safety and Quality Health Service Standards [39] benefits both nurses and organisations. For nurses, alignment of the HIRAID® education program with the National Safety and Quality Health Service Standards [39] made their contribution to safety and quality transparent to themselves but also others. For organisations, alignment of HIRAID® with the National Safety and Quality Health Service Standards [39] provided robust evidence to present to accreditation surveyors.

The College of Emergency Nursing Australasia (CENA) is the peak professional body representing emergency nurses across Australasia [40]. The CENA Practice Standards for the Specialist Emergency Nurse were first developed in 2008 [41] to identify areas of nursing practice and behaviours that articulate the unique characteristics of the speciality of emergency nursing, and recognise, promote and protect the discipline of emergency nursing [40]. The Practice Standards for the Specialist Emergency Nurse have nine domains: i) Clinical Expertise; ii) Communication; iii) Teamwork; iv) Resources and the Environment; v) Professional Development; vi) Leadership; vii) Lawful Practise; viii) Professional Ethics and ix) Research and Quality Improvement [40].

The CENA Practice Standards for the Specialist Emergency Nurse [40] are widely used in emergency nursing for staff appraisal, career planning and progression [42], by hospitals offering transition to specialty practice programs in emergency nursing [34], and universities offering postgraduate specialist emergency nursing courses [43]. It was therefore important that the HIRAID® education program reflected and acknowledged the unique and speciality specific knowledge and skills of emergency nurses.

### Phase 4: Behavioural diagnostic data and consultation with end users

The final phase in development of the HIRAID® education program was to use site specific behavioural diagnostics from the SW-cRCT clusters, and consultation with end users (emergency nurses, nurse educators, nurse managers, and nursing executives) through an iterative process [14]. The setting for Phase 4 was 29 EDs in New South Wales and Victoria, Australia [14]. By way of context there are 28 EDs in Victoria and 39 EDs in New South Wales [44]. The study EDs were organised into four clusters based on geography and governance structures (Table 1) and spanned rural, regional and metropolitan settings. For the purposes of the trial, each cluster had a site coordinator who was seconded from that cluster’s emergency nursing workforce so was known to staff. Each cluster also had a trial steering committee comprising hospital and ED key stakeholders including ED nurse managers and nurse educators [3].

**Table 1:** Characteristics of study sites.

Site	Context	ED patients per year	Admits via ED per year	Nurses	Description of Cluster	HIRAID® Commencement date
Cluster 1	10 rural, regional EDs	116,836	17,065	188	Spans 44,534km^2^ 200,000 + residents	09/11/2020
Cluster 2	12 rural, regional EDs	213,307	40,539	385	Spans 20,732km^2^ 350,000 + residents	06/10/2021
Cluster 3	4 metropolitan EDs	202,516	67,975	394	Spans 780km^2^ 946,000 + residents	08/08/2022
Cluster 4	3 metropolitan EDs	169,465	47,320	410	Spans 2816km^2^ 750,000 + residents	10/11/2022
**Total**	**29 EDs**	**702,124**	**172,899**	**1,377**	**45,060 km** ^**2**^ **2,246,000 + residents**	

**Abbreviations:** ED – emergency departments, km^2^ – kilometre, LHD – local health district.

HIRAID® commencement date = commencement of pre-HIRAID® behavioural diagnostics anonymous electronic surveys, surveys were open for 4 weeks at each site.

During the design of the HIRAID® implementation strategy, concurrent quantitative and qualitative data were collected at all study sites via an anonymous electronic survey to identify barriers and enablers to HIRAID® implementation: 670 nursing staff from 30 EDs completed the survey (58% response rate) and consent was implied by survey completion [4]. The electronic surveys commenced on the commencement dates listed in Table 1 and were open for 4 weeks. It should be noted that during the study design and set-up there were 30 EDs, however one ED from Cluster 1 was reclassified and lost ED status following completion of the behavioural diagnostics so did not go on to implement HIRAID®, hence 29 study sites.

The survey responses pertaining to the educational approach indicated that, to learn something new, nurses felt that face-to-face education (93.4%), hands on practice (91.1%), and the opportunity to ask questions (69.2%) were important [29]. Free text comments however raised concerns about lack of protected time or support for education (n = 24) [4]. Education was prominent in four of the six barriers (lack of knowledge about HIRAID®; lack of support to attend education or change practice; uncertainty about what to do; and lack of support or time for education) and one of three enablers (willingness to learn and adopt something new) to HIRAID® implementation [4].

## Results

The HIRAID® education program consisted of HIRAID® Provider (four-hours) and Instructor (one-day) Courses and was delivered using a train-the-trainer approach..

### HIRAID® provider course

The four-hour HIRAID® Provider Course consisted of pre-reading, a participant workbook, eLearning and an interactive facilitated workshop: each component was one-hour in duration. The HIRAID® Provider Course was informed by previous research; application of additional theoretical frameworks related to educational pedagogy, safety, quality, clinical governance and professional standards; and behavioural diagnostics data from the study sites. In the sections to follow, how each of these informed HIRAID® Provider Course design is detailed, followed by a description of the design of each element of the HIRAID® Provider Course learning resources.

The HIRAID® Provider Course was purposefully designed to be evidence-informed, provide structure for emergency nursing practice, and develop deep learning of core concepts and high-order thinking. To meet the intended learning outcomes, a suite of four scaffolded HIRAID® learning resources were developed: pre-reading, participant workbook, eLearning and interactive facilitated workshop.

The pre-reading was the Patient Assessment and Essentials of Care chapter from Australia’s only emergency nursing textbook [45]. The participant workbook, in fillable.pdf format that could be printed or completed electronically, guided participants through applying HIRAID® to two simple clinical scenarios in stable ‘patients’. In the first scenario, participants were provided with detailed prompts so they could use a structured but scaffolded approach to applying HIRAID®. On completion of the scaffolded scenario, participants worked through an integrated scenario without detailed prompts with the intention of seamless HIRAID® application. The workbook enabled participants to work at their own pace with relatively simple clinical scenarios but was asynchronous learning with written feedback. In the participant workbook, each element of HIRAID® was mapped to the National Safety and Quality Health Service Standards [39] and College of Emergency Nursing Australasia Standards for the Emergency Nursing Specialist [40] (Table 2) so participants connected use of HIRAID® with professional emergency nursing practice and national safety and quality standards.

**Table 2:** HIRAID® elements against the National Safety and Quality Health Service Standards [39] and CENA Practice Standards for the Specialist Emergency Nurse [40].

HIRAID® element	National Safety and Quality Health Service Standards	CENA Practice Standards for the Specialist Emergency Nurse
History	• Partnering with Consumers • Communicating for Safety	• Clinical Expertise • Communication
Infection risk	• Preventing and Controlling Infections	• Clinical Expertise • Communication • Resources and Environment
Red Flags	• Recognising and Responding to Acute Deterioration	• Clinical Expertise • Communication • Teamwork
Assessment	• Partnering with Consumers • Communicating for Safety • Comprehensive Care • Recognising and Responding to Acute Deterioration	• Clinical Expertise • Communication • Teamwork
Interventions	• Partnering with Consumers • Preventing and Controlling Infection • Communicating for Safety • Comprehensive Care • Recognising and Responding to Acute Deterioration	• Clinical Expertise • Communication • Teamwork
Diagnostic	• Partnering with Consumers • Communicating for Safety • Comprehensive Care • Recognising and Responding to Acute Deterioration	• Clinical Expertise • Communication • Teamwork • Resources and Environment • Lawful Practice
Reassessment	• Partnering with Consumers • Communicating for Safety • Comprehensive Care • Recognising and Responding to Acute Deterioration	• Clinical Expertise • Communication • Teamwork
Communication	• Communicating for Safety • Partnering with Consumers • Comprehensive Care	• Clinical Expertise • Communication • Teamwork • Resources and Environment • Lawful Practice

The eLearning reinforced, and built on, the pre-reading and participant workbook content by presenting two patient scenarios (one adult and one paediatric) of increased complexity, and with deterioration warranting timely medical assessment. Participants were required to think critically and prioritise the interventions required, and recognise clinical indicators for escalation, and were provided with examples of essential communication required during escalation of care for deteriorating patients. The eLearning modules were designed to be asynchronous self-directed learning but provided an active learning experience with immediate feedback, facilitating deeper learning and reflection on decision making. The eLearning modules also promoted participant reflection on **what** to communicate during routine communications and safety critical communication in the context of a deteriorating patient in preparation for the interactive facilitated workshop.

Finally, during an interactive facilitated workshop participants applied HIRAID® in real time to one or two of three patient scenarios (two adult and one paediatric) reflective of typical ED presentations. The patient scenarios again increased in complexity and featured deterioration requiring urgent medical assessment. The workshop was designed for synchronous active and collaborative learning with immediate instructor and peer feedback, and delivery in face-to-face or online formats. The workshop enabled participants to practice **how** they would communicate during routine communications, safety critical communication and during escalation of care of a deteriorating patient. Following completion of the workshop, participants were expected to work with clinical nurse consultants, clinical nurse educators, clinical facilitators, or senior nursing staff to apply the HIRAID® framework in conducting structured patient assessment and management in clinical practice.

**Fig 4:**
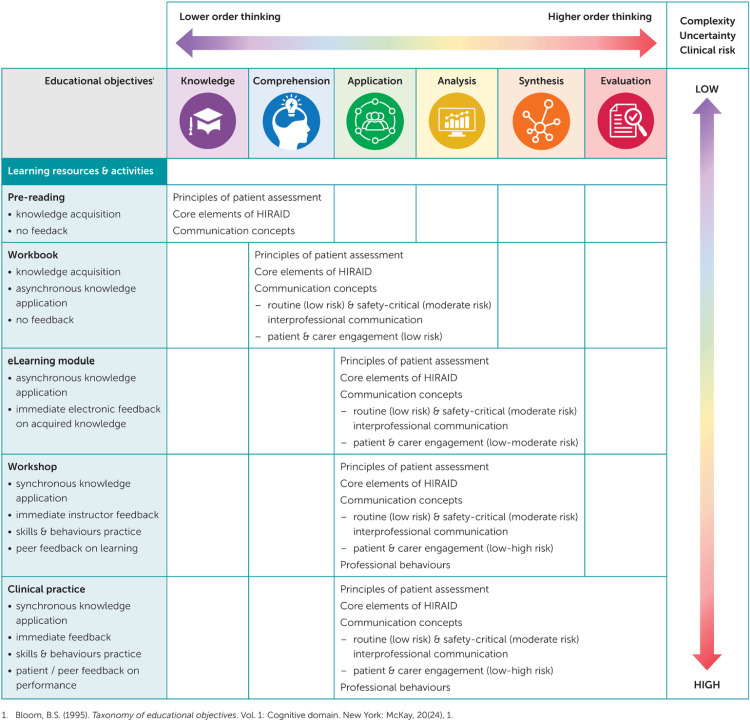
HIRAID® education program against Bloom’s taxonomy of educational objectives and decision complexity, uncertainty and clinical risk.

These learning resources were purposefully ordered to move participants through asynchronous to synchronous learning with increasing levels of feedback. The pre-reading and workbook equipped participants with requisite knowledge and core concepts, the eLearning provided a demonstration of HIRAID® use in realistic ED scenarios and provided real-time written and audio-visual feedback. These deliberate decisions were to scaffold learning and maximise use of the interactive workshop time for knowledge application rather than content delivery. The interactive workshop was designed to be facilitatory rather than didactic so that participants were actively engaged in knowledge application, received immediate and meaningful feedback, encouraged to think deeply, and develop and practice communication skills. A summary of the HIRAID® learning resources against Bloom’s taxonomy of educational objectives and decision complexity, uncertainty and clinical risk is shown in Fig 4. Each component of the HIRAID® education program was allocated one hour of continuous professional development by the Australian College of Nursing as recognition of learning.

### HIRAID® instructor course

The one-day HIRAID® Instructor Course was aimed at nurses with a role in providing HIRAID® Provider Courses in their EDs (such as clinical nurse educators, clinical nurse consultants, clinical facilitators, or senior nursing staff) and consisted of a HIRAID® facilitator manual, three PowerPoint® slide decks of patient scenarios, and an interactive facilitated instructor workshop. The facilitator manual provided HIRAID® Instructors with an overview of the intent, evidence base, and content of each element of the HIRAID® Provider Course, and equipped instructors with ground rules and participant expectations, guidance regarding facilitation of active and collaborative learning, and detailed lesson plans and slide decks for the Provider Course workshop. The facilitator manual provided HIRAID® Instructors with the intent, participant activities and facilitation tips for each slide in the slide decks, and was deliberately designed to decrease the workload of HIRAID® instructors and promote consistency of delivery (with local tailoring).

The HIRAID® Instructor workshop was facilitated by members of the research team and covered the evolution and evidence-base underpinning HIRAID®, all elements of the HIRAID® Provider Course, effective facilitation techniques and HIRAID® Instructor resources (HIRAID® facilitator manual and slide decks), and evidence-based strategies to effect behaviour change. It was reinforced to HIRAID® Instructors that their role was one of active facilitation by making it clear that the intent of the learning outcomes was to provide participants guidance about what they need to learn rather than a list of what facilitators had to teach. There were also clear expectations that participants attend the workshop having completed the pre-reading, workbook and eLearning; and that participants should drive their own learning.

### Education program delivery

A ‘train-the-trainer’ model was used to deliver the HIRAID® education program. Train-the-Trainer educational model is effective and cost-efficient and enables a multifaceted approach [46]. Train-the-trainer is commonly used in healthcare, and particularly nursing, and was the delivery approach in the 2017 HIRAID® implementation in four New South Wales EDs [9]. In preparation for HIRAID® implementation the study sites held HIRAID® Instructor Courses, facilitated by members of the research team. Fifteen HIRAID® Instructor Courses were held from February 2021 to March 2023 with 162 participants (Table 3).

**Table 3:** HIRAID® Instructor and Provider numbers per cluster.

Cluster	Number of EDs	ED nurse staffing	HIRAID® Instructors	Completed ≥1 element of HIRAID® Provider Course	Completed all 4 elements of HIRAID® Provider Course at 12 weeks
Cluster 1	10	188	34	230	133
Cluster 2	12	385	46	304	304
Cluster 3	4	394	35	468	370
Cluster 4	3	410	40	300	316
Total	29	1377	145	1302	1123

The HIRAID® Provider Course was deliberately designed to be modular with four discrete components (pre-reading, participant workbook, eLearning and interactive facilitated workshop) so that delivery could be tailored to the specific local needs. The study sites had different approaches to delivery of the HIRAID® Provider Course depending on study leave entitlements, backfill capacity, nurse staffing, number of HIRAID® Instructors and resources such as room availability. Some of the approaches included:

- Staff attending a four-hour session and completing all components of the HIRAID® Provider Course onsite. In the EDs that selected this option, the HIRAID® Instructors ran small group sessions for workbook completion and often created groups with a blend of experienced and less experienced nurses. The eLearning videos were displayed on a large screen so all participants viewed them at the same time, and were paused at major decision points aligned with each element of HIRAID®. The HIRAID® Instructors then facilitated peer-to-peer conversations before playing the video feedback; and then ran the workshop.
- Staff completing the pre-reading, workbook and eLearning off site (paid time) and attending an onsite one-hour workshop. In some EDs, the workshop was run during regular in-service education time and in other EDs there was a rolling series of workshops and staff rotated between working in the ED and attending the workshop.
- The way the workbook was operationalised also varied between EDs with some electing to have HIRAID® Instructors check workbook completion and in other EDs, staff completed a self-declaration that they had completed the pre-learning and workbook.

As of November 2023, over 1300 emergency nurses had completed the HIRAID® Provider Course (Table 3).

## Discussion

In this paper, the methods and processes used to develop and deliver a theory-informed education program to support behaviour change in emergency nursing are reported. A large programme of work has been undertaken to support development of the HIRAID® education program including previous research conducted over several years [8–11,18,19] and spanning simulation [8–11,18] and real-world testing [9–11]; use of behaviour change [21] and pedagogical theory [25–30]; alignment with national standards related to safety and quality of care [39] and emergency nursing [40]; and behavioural diagnostics from current study sites. The integration of theory, research, national standards and data from end users to substantial contribution to the scientific knowledge related to education programs for complex environments to support behaviour change.

### Benefits and challenges of using behaviour change theory

The COM-B model, Behaviour Change Wheel [21,22] and TDF [23,24] are highly effective in achieving behaviour change in a range of contexts. Some view theory-informed approaches as overly prescriptive [47], therefore engaging in co-design and integrating clinician innovation and creativity was crucial in designing the HIRAID® education program. Theory-driven behaviour change takes time, which can conflict with a desire for rapid change, particularly if related to an adverse event, clinical risk, or achieving compliance.

Some elements of the Behaviour Change Wheel translated more easily into education than others. The target behaviour was well defined (use of HIRAID®) and the behavioural diagnostics provided a clear indication of what needed to change, and the barriers to that change. The Behaviour Change Wheel provides guidance regarding intervention options, but how the intervention functions were presented to clinicians and key stakeholders was important. Language such as ‘restriction’, ‘coercion’, ‘incentivisation’ may be perceived as negative if the theoretical context in which these terms were used was unclear. There was little opportunity to change policy categories as many facets of organisational governance are pre-determined, for example, compliance with National Safety and Quality Health Service Standards [39]. Identifying content and implementation options (behaviour change techniques and mode of delivery) was theoretically straightforward, but operationalising some techniques and delivery was complicated. Finally, HIRAID® education was part of a broader implementation strategy, however it was challenging to stop people thinking the HIRAID education was the implementation strategy.

### Benefits and challenges of using educational theory in nursing education

Variability in pedagogy carries the risk of nursing educational programs failing to achieve their desired outcomes [6]. The use of pedagogical theory [25–30] in the HIRAID® education program ensured consistency of an evidence-based educational approach with clear intended learning outcomes at the core of all learning activities. The HIRAID® Instructor Course (including the HIRAID® Instructor manual) focused on practical content using an evidence-informed approach to learning and teaching in nursing education. The HIRAID® education program demonstrates that a sound educational methodological approach to nursing education can bridge the evidence-practice gap in real-time and real-life clinical settings.

Although clinical nurse educators were a target audience for the HIRAID® Instructor Course, the instructor course was purposefully designed to be inclusive of nursing staff not in formal education roles, thus built education capacity at the study sites. The train-the-trainer model was a cost-effective, sustainable method of education delivery for hard to reach and time restricted populations (such as nurses) in settings with limited resources (such as emergency departments) [48]. The nature of emergency nurses’ work (shiftwork, unpredictable workloads) can make education access challenging and most EDs have limited nurse educators. The train-the-trainer model overcame these challenges whilst building educational capacity for nurses not in formal education roles. Nurse educators can use the methods and processes outlined in this paper to inform the design and delivery of education components of practice change initiatives.

### General challenges

There were a range of general challenges in undertaking this research. Significant time investment was required to prepare HIRAID® Instructors and provide both instructors and nurses dedicated time to deliver or complete the HIRAID® Provider Course in the context of high and unpredictable workloads, time pressures, and staff shortages. The development and delivery of theory-informed education program to support behaviour change in emergency nursing was innovative, and challenged some traditional nursing education norms so stakeholder management was critical. Finally, this work was complicated by the COVID-19 pandemic and a series of natural disasters (bushfires in Cluster 1 and floods in Cluster 2 that disabled several hospitals in that district).

### Strengths and limitations

In this paper, the methods and processes used to develop and deliver a theory-informed education program to support behaviour change in emergency nursing are described. The strengths of this work are the contribution to implementation science and a practical example of situating education within a broader multi-faceted implementation strategy. The theory-informed approach, integration of national and professional safety and quality standards, and engagement with end-users are additional strengths. The key limitations of the approach to develop and deliver the HIRAID® education were that it was resource and time intensive. This paper does not include any participant feedback as it is yet to be collected, but end-user experience will be reported as one of the trial outcomes.

## Conclusion

Development of an emergency nursing education program as part of a multi-faceted implementation strategy aimed at behaviour change using a robust theoretical approach is possible. It is also feasible to deliver a theory-informed education program for nurses working in dynamic environments such as the ED. Although HIRAID® education program development was complex, it resulted in a structured and feasible method of delivery. The approach described in this paper provides a blueprint for other researchers aiming to change behaviours in complex clinical settings.

## Registration details

ACTRN12621001456842 (Australian New Zealand Clinical Trials Registry (ANZCTR)).
